# Traumatic stress and psychological functioning in a South African adolescent community sample

**DOI:** 10.4102/sajpsychiatry.v23i0.1008

**Published:** 2017-03-10

**Authors:** Karl D. Swain, Basil J. Pillay, Wendy Kliewer

**Affiliations:** 1 Department of Behavioural Medicine, University of KwaZulu-Natal, South Africa; 2 Department of Psychology, Virginia Commonwealth University, United States

## Abstract

**Background:**

Traumatic stress may arise from various incidents often leading to posttraumatic stress disorder (PTSD). The lifetime prevalence of PTSD is estimated at 1% – 2% in Western Europe, 6% – 9% in North America and at just over 10% in countries exposed to long-term violence. In South Africa, the lifetime prevalence for PTSD in the general population is estimated at 2.3%.

**Aim:**

To examine the prevalence of posttraumatic stress symptomatology and related psychological functioning in a community sample of adolescents.

**Setting:**

Low-socioeconomic communities in KwaZulu-Natal.

**Methods:**

Home interviews with adolescents and their maternal caregivers were used to collect the data using standardised instruments. Adolescents completed the Trauma Symptom Checklist for Children; Children’s Depression Inventory; Children’s Somatization Inventory; and Revised Children’s Manifest Anxiety Scale. The Child Behaviour Checklist was completed by the caregivers. The sample comprised Grade 7 (*n* = 256) and Grade 10 (*n* = 68) learners. Sixty-five percent of the sample was female, and ages ranged from 9 to 18 (*M* = 13.11, s.d. = 1.54).

**Results:**

Almost 6% of the sample endorsed PTSD and an additional 4% of the participants had clinically significant traumatic stress symptomatology. There was a significant, large, positive correlation between posttraumatic stress and anxiety, and medium positive correlations between posttraumatic stress and depression and somatic symptoms.

**Conclusion:**

Posttraumatic stress symptomatology can be debilitating, often co-occurring with symptoms of depression, anxiety and somatic complications. This may lead to long-term academic, social and emotional consequences in this vulnerable group.

## Introduction

Traumatic stress may arise from a variety of incidents, including artificial or natural disasters such as earthquakes and motor vehicle accidents or community violence such as gang violence, neighbourhood gun warfare, rape, school violence and victimisation.^1,2^ Traumatic stress severely impacts personal functioning, interpersonal relationships and employment, and it is associated with a variety of other psychological conditions, including posttraumatic stress disorder (PTSD), anxiety, depression and somatisation.^1,2^ However, many individuals exposed to trauma experience symptoms of PTSD that do not always meet diagnostic thresholds.^2^ These people, who suffer debilitating symptoms at a sub-threshold level, are considered to have posttraumatic stress symptomatology (PTSS) for the purposes of this study. In this study, both PTSD and PTSS are considered.

The global lifetime prevalence of PTSD is estimated to be 1% – 2% in Western Europe, 6% – 9% in North America and just over 10% in countries exposed to long-term violence. This widespread difference in prevalence is attributable to the variation in the exposure to traumatic events according to these authors,^3^ but it is well-known that trauma exposure is only one, albeit necessary, factor in the development of PTSD. In the USA, prevalence rates for the general population were 5.0% for males and 10.4% for females. In Europe, the general population prevalence rates range from 0.5% for males and 0.7% for females^4^ in Iceland, to 0.56% in Spain, 0.76% in Belgium, 3% in the UK (0%) and 3.3% in the Netherlands.^5^ The lifetime prevalence rates of PTSD in the general South African population have been found to be 2.3% for all ages and 1.8% for ages between 18 and 34 with the 12-month prevalence rate being 0.6% – 0.7%; sex, age and education were largely unrelated to PTSD risk.^6^

Interpersonal violence prevails in South Africa with mortality rates reaching seven times that of the global rate.^7^ Interpersonal violence injuries are the second most dominant source of loss of healthy life, representing 14.3% of all disability-adjusted life years in South Africa in 2000.^7^ Furthermore, South Africa has one of the highest motor vehicle accident rates in the world^8^ and road traffic injuries are double that of the global rate.^7^

In a South African-based rural study^9^ that linked violence exposure to health outcomes in children, 67.0% had directly or vicariously experienced a traumatic event with 8.4% having a PTSD diagnosis. A study^10^ conducted in a Khayelitsha school and children’s home found that all participants had reported exposure to indirect violence: 95.0% had witnessed violence and 56.0% had personally experienced violence, with 21.7% meeting the criteria for PTSD. In another study^11^ which looked at 2041 boys and girls from various schools in Cape Town and Nairobi, 80% of the sample reported exposure to severe trauma. The South African cohort reported witnessing violence (58.0%), physical assault by family (14.0%) and sexual assault (14.0%) – 22.2% met the criteria for PTSD and an additional 12.0% for sub-diagnostic PTSD. This study also noted no sex differences in PTSD symptom reporting.

PTSD is associated with psychiatric comorbidity, most notably substance use disorder, major depression, anxiety and somatic disorders.^12,13^ Major depressive disorder as a comorbid condition with PTSD ranges from 30% to 50%^1,11,12^ and is significantly correlated with exposure to violence.^14^ Anxiety disorders are prevalent psychiatric disorders in childhood and adolescence, with 10% – 15% meeting the diagnostic criteria for an anxiety disorder.^15^

PTSD and its associated sequelae are, therefore, a major public health concern in South Africa,^16^ particularly for youth who are understood to be experiencing high levels of trauma stemming from discrimination, abuse, interpersonal violence and traumatic events, often against a backdrop of socioeconomic disproportion, reflecting the widening gap between the rich minority and the large proportion of poor in South Africa.^16,17^

Many of the studies reviewed have employed clinical samples, with little work focusing on local community samples. Furthermore, most community-based studies in KwaZulu-Natal focused on single conditions and did not investigate co-occurrences of psychiatric symptoms. The aim of this study was to examine the prevalence of traumatic stress symptomatology and related psychological functioning in a community sample of adolescents in low-socioeconomic areas of KwaZulu-Natal.

## Ethical Considerations

Ethical approval was obtained from the Biomedical Research Ethics Committee of UKZN. The KwaZulu-Natal Department of Education approved the study and consent was obtained from the relevant school principals. Participation in the study was voluntary and participants could withdraw at any stage. In order to compensate and show appreciation for their time, shopping vouchers to a value approved by the ethics committee were provided to the participants.

## Research methods and design

### Study design

A cross-sectional study design was used. The sample was dichotomised into participants with posttraumatic symptomatology (PTS+) and those without (PTS-) in order to examine the prevalence of traumatic stress symptomatology and related psychological functioning in this sample.

### Setting

The study was conducted within low-socioeconomic communities in Durban, KwaZulu-Natal. Government census statistics were used to identify low-socioeconomic communities based on levels of crime, education and income.^18,19^

### Study population and sampling strategy

Participants were maternal caregivers (*N* = 324) and their adolescents who were either in Grade 7 (*n* = 256) or Grade 10 (*n* = 68). Sixty-five percent of the sample was female. The majority of the participants were black African (55.00%), followed by Indian (23.44%), mixed race (14.69%) and white people (6.88%). The age of the participants ranged from 9 to 18 years (*M* = 13.11, s.d. = 1.54).

### Data collection

Principals of identified schools were approached to participate in the study and learners were addressed in their classrooms based on a standardised script. Information packs, comprising a formal letter addressed to their maternal caregiver that described the study, assent forms and consent forms, were distributed to the learners. Caregivers and adolescents who agreed to participate provided written consent and assent, respectively, and were contacted to arrange for the home-based interviews. Each adolescent and their maternal caregiver were interviewed separately. At any given time four research assistants were trained to conduct the interviews.

### Instruments

Several scales were used to collect the data. The Child Behaviour Checklist (CBCL) was completed by the maternal caregivers and all other scales were completed by the adolescents.

#### Trauma Symptom Checklist for Children

The Trauma Symptom Checklist for Children (TSCC)^20^ is a 54-item self-report measure that evaluates symptoms of posttraumatic stress and related psychological symptomatology in children aged 8–16 years and standardised on a large sample of racially and economically diverse children.^20^ The reliability analyses in the normative sample yielded acceptable internal consistencies ranging from the mid-to-high 80s for all scales, except the sexual concerns scales. This study used the posttraumatic stress and anxiety sub-scales from the TSCC. The Cronbach alphas in this study were α = 0.97 (posttraumatic stress) and α = 0.83 (anxiety).

#### Children’s Depression Inventory

The Children’s Depression Inventory (CDI)^21^ is a 27-item self-report measure of cognitive, affective and behavioural symptoms of depression for school-aged children and adolescents. The CDI has good sensitivity and specificity, as well as high test-retest reliability and internal consistency coefficients ranging from 0.59 to 0.89.^21,22^ The CDI was found to be highly reliable in this study (α = 0.85).

#### Children’s Somatization Inventory

The Children’s Somatization Inventory (CSI)^23^ self-report instrument evaluates the occurrence of somatisation symptoms in children and adolescents. Previous research^23^ has supported the reliability of the CSI with a Cronbach alpha of 0.88. The internal consistency in this study was Cronbach α = 0.91.

#### Revised Children’s Manifest Anxiety Scale

The Revised Children’s Manifest Anxiety Scale (RCMAS) is a 37-item self-report instrument designed to measure emotions and physical symptoms of anxiety in children and adolescents aged between 6 and 19 years.^24^ Previous research^24^ has reported high coefficients alpha for the total anxiety scale (α = 0.80). In this study α = 0.90 (total anxiety) and α = 0.67 (physiological anxiety).

#### Child Behaviour Checklist

The CBCL^25^ was completed by the maternal caregiver and contains a series of 113 items that help assess a child’s behavioural and emotional problems over the past 3 months. Syndromes on the CBCL are classified into one of eight areas as follows: anxious/depressed, withdrawn, thought problems, attention problems, somatic complaints, social problems, aggressive behaviour and delinquent behaviour. Adequate reliability indices are reported^25^ and in this study the reliability for the anxiety-depression sub-scale was α = 0.81 and α = 0.77 for the somatic complaints sub-scale.

### Data analysis

The Statistical Package for Social Sciences version 22 (SPSS 22) was employed for the analyses of data. The sample was dichotomised into participants with PTS+ and those without (PTS-) based on a *t*-score of ≥ 60 on the PTS sub-scale of the TSCC.^20^ A *t*-score of ≥ 65 on this scale is indicative of PTSD. The author^20^ suggests that scores between 60 and 64 are regarded as clinically significant and indicative of PTSS. The PTS+ cohort is therefore composed of those with a *t-*score of ≥ 60. Descriptive statistics as well as correlations between the study variables were undertaken. Independent *t*-tests were conducted to compare means and standard deviation scores for PTS+ and PTS- groups, to evaluate differences between the groups on scores for posttraumatic stress, anxiety, depression and somatisation. A series of two-way ANOVAs were conducted to compare the PTS+ and PTS- groups by race, grade and sex.

### Results

Descriptive statistics (Table 1) as well as correlations between the study variables (Table 3) were undertaken. Fifty-five percent of the youth were not living with two parents. In terms of the maternal caregivers, 54.0% had less than a high school level of education. Household income was reported as follows: 5.2% (less than R500 per month), 12.3% (R501 – R1500 per month), 13.3% (R1501 – R2500 per month), 9.9% (R2501 – R3500 per month), 11.7% (R3501 – R4500 per month), 8.6% (R4501 – R5000 per month) and 34.6% (R5001+ per month). Furthermore, 56.2% reported that this income was stable, whilst 43.8% noted this income was unstable and changed monthly.

**TABLE 1:** Descriptive statistics for demographical variables for the PTS+ group and PTS- group.

Variable	*N*	%	PTS+				PTS-				*F*	*p*
	
			*n*	%	*M*	s.d.	*n*	%	*M*	s.d.		
**Race**												
Black African	176	55.00	17	53.13	19.20	3.90	159	55.21	4.32	3.84	0.44	0.72
Mixed race	47	14.69	4	12.5	18.00	2.94	43	14.93	4.03	3.99		
Indian	75	23.44	6	18.75	17.28	1.06	69	23.96	4.33	3.75		
White people	22	6.88	5	15.63	18.00	2.35	17	5.9	4.30	3.70		
Total	320	100.	32	100	18.50	3.19	288	100	4.28	3.81		
**Grade**												
Grade 7	252	78.75	21	65.63	18.19	3.27	231	80.21	4.14	3.76	1.13	0.29
Grade 10	68	21.25	11	34.37	19.09	3.12	57	19.79	4.84	4.02		
Total	320	100	32	100	18.50	3.19	288	100	4.28	3.81		
**Sex**												
Male	112	35	11	34.34	17.18	3.71	101	35.07	4.26	3.46	1.90	0.17
Female	208	65	21	65.63	19.19	2.73	187	64.93	4.29	4.00		
Total	320	100	32	100	18.50	3.19	288	100	4.28	3.81		

* *p* < 0.01

s.d., standard deviation.

On the TSCC, 19 participants (5.94%) had *t*-scores of ≥ 65 and 13 participants (4.06%) had posttraumatic stress symptoms, with sub-diagnostic (*t*-scores) between 60 and 64. Thus, the total PTS+ group consisted of 32 participants (10% of the sample). Independent *t*-tests comparing age for the PTS+ and PTS- groups showed a significant difference between the PTS+ (*M* = 13.69, s.d. = 1.53) and the PTS- (*M* = 13.05, s.d. = 1.53, *t*(318) = -2.24, *p* = 0.02) groups indicating that the PTS+ group was significantly older. Independent *t*-tests (Table 2) comparing the PTS+ and PTS- groups revealed that the PTS+ group reported significantly more anxiety, depression and somatic symptoms.

**TABLE 2:** Comparison of means and standard deviation scores for PTS+ and PTS- groups for PTS, anxiety, depression and somatisation.

Variable	PTS+		PTS-		*t*

	*M*	s.d.	*M*	s.d.	
1. PTS	18.50	3.19	4.28	3.81	−23.41**
2. Anxiety (TSCC)	12.15	5.26	3.29	3.34	−13.32**
3. CDI	15.47	7.92	7.69	6.16	−5.38**
4. CSI	22.44	14.31	10.35	10.15	−6.1**
5. Physiological anxiety (RCMAS)	4.13	2.38	1.83	1.78	−6.68**
6. Total anxiety (RCMAS)	16.01	6.21	8.07	5.82	−6.9**
7. CBCL Anxiety-depression	5.53	5.28	4.76	4.57	−0.87
8. CBCL Somatic complaints	3.75	4.13	3.49	3.36	−0.41

*N* = 320; *p < 0.01;

** *p* < 0.001

s.d., standard deviation.

PTS+, with posttraumatic symptomatology; PTS-, without posttraumatic symptomatology; TSCC, Trauma Symptom Checklist for Children; CDI, Children’s Depression Inventory; CSI, Children’s Somatization Inventory; RCMAS, Revised Children’s Manifest Anxiety Scale; CBCL, Child Behaviour Checklist.

The relationship between PTSS and the other study variables of anxiety, depression and somatic symptoms is represented in Table 3. There was a significant, positive correlation between posttraumatic stress and anxiety measured by the TSCC, physiological anxiety and total anxiety measured by the RCMAS, as well as youth-rated depressive and somatic symptoms. Interestingly, PTSS was not correlated with caregiver’s ratings of anxiety, depression or somatic symptoms on the CBCL. Thus, a cross-rater effect was observed in that there was a clear discrepancy between what was being reported by the youth and their maternal caregivers in terms of the symptoms experienced by the adolescents.

**TABLE 3:** Correlations between posttraumatic stress, anxiety, depression, and somatisation.

Variable	1	2	3	4	5	6	7	8
1. PTS	-	0.83**	0.49**	0.41**	0.52**	0.57**	0.10	0.01
2. Anxiety (TSCC)		-	0.47**	0.44**	0.53**	0.60**	0.11*	0.04
3. CDI			-	0.52**	0.65**	0.69**	0.17**	0.06
4. CSI				-	0.62**	0.60**	0.07	0.02
5. Physiological anxiety (RCMAS)					-	0.84**	0.08	0.03
6. Total anxiety (RCMAS)						-	0.09	0.01
7. Anxiety-depression (CBCL)							-	0.48**
8. Somatic complaints (CBCL)								-
M	5.70	4.18	8.46	11.53	2.04	8.83	4.83	3.56
s.d.	5.69	4.45	6.73	11.17	1.97	6.31	4.62	3.48

Sample size ranged from 318 to 324 because of missing data.

s.d., standard deviation.

PTS, Posttraumatic Stress Sub-Scale of TSCC; TSCC, Trauma Symptom Checklist for Children; CDI, Children’s Depression Inventory; CSI, Children’s Somatization Inventory – Short Form; RCMAS, Revised Children’s Manifest Anxiety Scale.

* *p* < 0.05;

** *p* < 0.01

Table 4 presents the two-way ANOVAs for race, grade and sex in those with PTSS and those without. A two-way between-groups analysis of variance was conducted to explore the impact of race on levels of PTS scores. There was no statistically significant main effect for race (*F* [3, 312] = 0.44, *p* = 0.72). A two-way between-groups analysis of variance was conducted to explore the impact of grade on levels of PTS scores. There was no statistically significant main effect for school grade (*F* [1, 316] = 1.13, *p* = 0.29). A further two-way between-groups analysis of variance was conducted to explore the impact of sex and PTS groupings on levels of PTS scores. There was no statistically significant main effect for sex (*F* [1, 316] = 1.90, *p* = 0.17).

**TABLE 4:** ANOVAs for race, grade and sex on PTS, anxiety, depression, and somatisation.

Variable	Race		Grade		Sex	
		
	*F*	*p*	*F*	*p*	*F*	*p*
1. PTS	0.44	0.72	1.13	0.29	1.90	0.17
2. Anxiety (TSCC)	1.47	0.22	0.11	0.74	10.92	0.00***
3. CDI	1.17	0.32	1.20	0.27	2.53	0.11
4. CSI	0.64	0.59	0.32	0.57	3.62	0.06
5. Physiological anxiety (RCMAS)	2.10	0.10	5.01	0.03*	1.62	0.20
6. Total anxiety (RCMAS)	2.34	0.07	8.11	0.01**	2.74	0.10
7. CBCL anxiety-depression	1.53	0.21	0.20	0.66	0.69	0.41
8. CBCL somatic complaints	0.58	0.63	4.99	0.03*	2.14	0.15

*N* = 320;

* *p* < 0.05;

** *p* < 0.01;

*** *p* < 0.001

Df: Race = 3; Grade = 1; Sex = 1.

PTS, posttraumatic symptomatology; TSCC, Trauma Symptom Checklist for Children; CDI, Children’s Depression Inventory; CSI, Children’s Somatization Inventory; RCMAS, Revised Children’s Manifest Anxiety Scale; CBCL, Child Behaviour Checklist.

Multiple two-way ANOVAs were conducted considering the impact of race, grade and sex on the other study variables of anxiety, depression and somatic symptoms (see Table 4) with regard to PTS. Grade 10 participants reported higher scores (*M* = 2.70, s.d. = 2.20) on the physiological anxiety sub-scale than Grade 7 participants (*M* = 1.89, s.d. = 1.87). A similar trend was seen on the RCMAS total anxiety scores (Grade 10: *M* = 11.53, s.d. = 6.77; Grade 7: *M* = 8.14, s.d. = 6.00). Female participants (*M* = 9.16, s.d. = 6.58) reported significantly higher levels of total anxiety (RCMAS) than males (*M* = 8.32, s.d. = 5.80). Similarly, female participants scored significantly higher scores on the TSCC anxiety sub-scale (*M* = 4.41, s.d. = 4.74) than males (*M* = 3.75, s.d. = 3.84). Females (*M* = 11.85, *SD* = 11.18) in the overall sample reported marginally significantly higher somatic symptoms than males (*M* = 11.02, s.d. = 11.30).

## Discussion

This study investigated the relationship between traumatic stress and anxiety, depression and somatisation in a multi-racial community sample. Ten percent of the adolescents in this study endorsed responses on the TSCC that indicated clinically significant PTSS. Almost 6.0% of the youth had PTSD (*t*-scores ≥ 65). This result is lower than other local studies, in which 8.4% of the sample had PTSD. Another study of adolescents from schools across Cape Town^11^ indicated that 22.2% of this sample had PTSD, with an additional 12.0% reaching a sub-diagnostic threshold, and a further study among adults^6^ indicated a prevalence of 2.3%. Our results are similar to research in countries exposed to long-term violence^3^ that indicate PTSD prevalence rates of over 10.0%.

It was hypothesised that traumatic stress levels would be positively correlated with levels of anxiety, depression and somatic symptoms. This study demonstrated a large positive correlation between traumatic stress and anxiety, and medium positive correlations between posttraumatic stress and depression and somatic symptoms, which is similar to other research findings.^26,27,28^ Our study supports the notion that PTSD is associated with comorbidities of depression and anxiety.^1,29,30,31^ Interestingly, there appeared to be a cross-rater effect as noted above, in that the level of adolescent self-reported symptomatology and that reported by their maternal caregivers varied greatly. As Table 3 shows, although both adolescents and caregivers reported on the various symptomatology, the reports from the caregivers were not highly correlated. Such discrepant patterns have been observed in various studies^32^ and a meta-analysis^33^ supported a robust finding of discrepancy between different raters of emotional and behavioural symptoms. This may support the notion that adolescents may be more cognisant of their internal states and symptoms, than adults such as caregivers and teachers. Hence, such adults may not be accurately or timely identifying the suffering of youth. As such, interventions need to be designed to assist caregivers in identifying disorders or symptoms so that timely intervention may be sought.

No statistically significant main effect for sex on PTS scores was found suggesting that sex was found to be unrelated to PTSD risk which is supported by other studies.^11,34^ However, although sex was unrelated to PTS scores, female participants had significantly higher anxiety and somatic symptoms. Factors including biological differences, differences in symptom appraisal and assessment, and most importantly socialisation and social roles may contribute to such sex differences.^35^ Females tend to report more frequent, numerous and intense somatic symptoms than males in both medical and community samples because of factors such as differences in symptom labelling or description, acknowledgement of discomfort and sensation perception.^35^ Furthermore, anxiety disorders are 2–3 times more prevalent in females and these disorders have somatic components, which may also contribute to higher reporting of somatic problems in females.^35^

### Implications for clinical practice and policy

PTSD can be a debilitating disorder and may often have long-term academic, social and emotional consequences for an individual.^36^ Some studies^37,38^ show that exposure to traumatic events are associated with decreased IQ, lower school grade averages, higher school absenteeism rates and even decreased rates of high school completion. Such evidence supports the need for adequate intervention and prevention strategies. However, in South Africa many individuals do not have adequate access to health care resources, and adolescents are often being taken care of within disjointed family constitutions where basic needs may become prioritised over mental health issues because of poverty and other social issues.

It is paramount that youth mental health status needs be addressed, as PTSS often has delayed comorbidity and is associated with several problems outlined above. As noted in other work,^39^ trauma exposure and PTSD, whilst prevalent amongst adolescent psychiatric inpatients, are frequently under-identified in routine clinical practice. The development of norms and better screening tools for local populations as well as the early detection by involving teachers will help identify at-risk children who may be referred to appropriate mental health workers for assistance.^36^

### Study limitations and directions for future research

A major limitation of this study is the cross-sectional design, which did not allow for the analysis of changes in symptomatology over time and their associated comorbidities. A further limitation is the exclusive reliance on self-reported psychometric inventories, the reliability of which may be affected by inattention, flawed recall or deliberate distortion.^40^ Augmenting these measures with clinical and collateral interviews could therefore be beneficial in future studies, especially since the discussed cross-rater effect was observed in that there was a clear discrepancy between what was being reported by the youth and their caregivers in terms of some symptomatology. Despite these limitations, the findings augment the existing literature by tracking community levels of PTSS and support the co-occurrence and relationship of traumatic stress and anxiety, depressive and somatic symptoms.

## Conclusion

This community-sample study concluded that 10% of adolescents endorsed clinically significant PTSS, with almost 6% having PTSD. The results showed a correlation between traumatic stress and anxiety as well as between posttraumatic stress and depression and somatic symptoms. Females reported higher anxiety and somatic symptoms (Tables 1–4).
